# Emerging leptospirosis in urban Sydney dogs: a case series (2017–2020)

**DOI:** 10.1111/avj.13148

**Published:** 2022-01-25

**Authors:** C Griebsch, N Kirkwood, MP Ward, W So, L Weerakoon, S Donahoe, JM Norris

**Affiliations:** ^1^ Sydney School of Veterinary Science University of Sydney Sydney New South Wales 2006 Australia

**Keywords:** Australia, canine, *Leptospira*, leptospirosis, outbreak

## Abstract

Canine leptospirosis has not been reported in the Sydney dog population since 1976. However, between 2017 and 2020, leptospirosis was confirmed in 17 dogs, five of which were known to hunt rodents. Dogs infected between 2017 and 2019 lived within a 3 km radius in the Inner City of Sydney (n = 11). In 2020, cases emerged across a broader area of Sydney; Inner City (n = 1), Inner West (n = 3), Lower North Shore (n = 1) and Upper North Shore (n = 1). The disease was characterised by severe hepatorenal involvement resulting in an unusually high case fatality rate (88%). In conjunction with supportive clinical signs, diagnosis was confirmed by real‐time PCR on whole blood (n = 1), kidney (n = 1), urine (n = 4), whole blood and urine (n = 9) or by seroconversion (n = 3). Antibody titres determined by Microscopic Agglutination Test (MAT) to *Leptospira* serovars were measured in 12 dogs: seven were positive for serovar Copenhageni, one was positive for serovar Hardjo, three were negative for all serovars, likely due to insufficient time for seroconversion before death and one had a low positive titre (1/50) for serovars Australis and Robinsoni. This sudden emergence of a highly fatal disease in pet dogs in Sydney has led to the introduction of *Leptospira* vaccination protocols for dogs living in inner Sydney using a monovalent vaccine containing serovar Copenhageni. The success of this vaccination program will require ongoing research to understand the emergence of leptospirosis in this region and the serovars involved.

**Abbreviations**
AKI: acute kidney injury
aPTT: activated partial thromboplastin time
DIC: disseminated intravascular coagulation
IRIS: International Renal Interest Society
IVF: intravenous fluid
LPHS: leptospirosis pulmonary haemorrhage syndrome
MAT: Microscopic Agglutination Test
PT: prothrombin time

Leptospirosis is caused by a motile aerobic spirochete bacterium of the genus *Leptospira*. In dogs, the most pathogenic serovars cause vasculitis leading to tissue injury, including acute kidney and hepatic disease. Importantly, several serovars are zoonotic. Rodents are the most common reservoir hosts for *Leptospira* genotypes and shed leptospires in their urine.^1^, ^2^ Dogs become infected by contact with urine or indirectly via contaminated environments.^1^ Following infection, bacteraemia occurs lasting around 10 days followed by vasculitis, organ ischaemia and invasion of the kidneys and liver, resulting in shedding of leptospires into urine.^3^ Leptospirosis should be suspected in dogs with nonspecific clinical signs (lethargy, vomiting, diarrhoea, haemorrhages, conjunctivitis), consistent clinicopathological abnormalities (azotaemia, hyperbilirubinaemia, increased liver enzymes, glucosuria) and risk factors (rodent contact, exposure to contaminated environments).^1^ Fatality rates of 18% to 55% have been previously described in cases from Australia,^4^, ^5^ Europe^6^, ^7^, ^8^, ^9^, ^10^ and the USA.^11^, ^12^ Diagnosis can be achieved by PCR on blood or urine prior to antibiotic administration or markedly elevated antibody levels to specific *Leptospira* serovars (Microscopic Agglutination Test [MAT]) or seroconversion (4‐fold rise in antibody titre).^1^, ^6^, ^8^, ^9^ While case definition varies across jurisdictions, these criteria are used to confirm a diagnosis of human leptospirosis in countries with a high leptospirosis incidence (New Zealand,^13^ USA^14^).

Prevention is achieved by vaccination and limiting contact with sources of infection.^3^ There is one registered vaccine in Australia (Protech® C2i, Boehringer Ingelheim) containing serovar Copenhageni.^15^ Historically, dogs in Sydney have not been vaccinated due to apparent absence of disease. In the most recent limited serosurvey of 956 healthy dogs in Australian animal shelters in 2004, seroprevalence of 2.4% was estimated in New South Wales in 431 dogs.^16^ Copenhageni was the most prevalent serovar (5/10 dogs). Earlier seroprevalence studies in dogs with no known history of exposure showed seroprevalence of 9.8% in dogs in New South Wales and Victoria (501 dogs) in 1990^17^ and 6.8% in Sydney (600 dogs) in 1972,^18^ and Copenhageni was the most prevalent (16/49 and 30/41, respectively).

No literature describing clinical leptospirosis in New South Wales has been published since 1976.^19^, ^20^, ^21^ In this study, we characterise clinicopathological findings, diagnostic imaging and clinical outcomes in dogs diagnosed with leptospirosis in a recent Sydney outbreak with a high fatality rate.

## Materials and methods

Medical records of cases presented between December 2017 and June 2019 were retrospectively reviewed. From July 2019, cases were enrolled prospectively. Cases were identified following referral or direct contact from referring veterinarians after a leptospirosis alert was issued across Sydney. Leptospirosis was suspected in dogs with consistent clinical and clinicopathological findings (azotaemia, hyperbilirubinaemia, elevated liver enzymes). Diagnosis was confirmed by positive PCR on blood, urine or kidney tissue (IDEXX or Vetnostics laboratories), the presence of *Leptospira*‐shaped organisms in the kidneys identified with silver stain (Warthin‐Starry) or via seroconversion (4‐fold increase in MAT titre) or a MAT titre ≥1/800 in nonvaccinated dogs (WHO Leptospirosis Laboratory, Public and Environmental Health, Department of Health, Cooper Plains, Queensland; 23 serovar assay testing for serovars Arborea, Australis, Bataviae, Bulgarica, Canicola, Celledoni, Copenhageni, Cynopteri, Djasiman, Grippothyphosa, Hardjo, Icterohaemorrhagiae, Javanica, Kremastos, Medanensis, Panama, Pomona, Robinsoni, Shermani, Swajizak, Tarassovi, Topaz, Zanoni). Real‐time PCR was used at both reference veterinary diagnostic laboratories. Both are National Association of Testing Authorities accredited with all tests run with quality controls.

Medical records were reviewed for signalment, history, vaccination status, physical examination findings, results of haematology, serum biochemistry, coagulation profiles (prothrombin time [PT], activated partial thromboplastin time [aPTT]), urinalysis, diagnostic imaging, urine output, blood pressure measurements, treatment regime, outcomes and postmortem findings.

Cases were classified based on organ involvement (renal, hepatic, pulmonary, haemorrhagic).^9^ Acute kidney injury (AKI) was defined as documented AKI (historical, clinical, laboratory, imaging evidence), oliguria (<1 mL/kg/h) or anuria (no urine production) and progressive increase in creatinine concentration of >26.4 μmol/L within 48 h according to International Renal Interest Society (IRIS) guidelines.^22^ Severity of AKI was based on the IRIS grading system.^22^ The lowest urine output during hospitalisation was reported. Hepatic involvement was defined as presence of elevated liver enzymes and hyperbilirubinaemia and classified as mild (serum bilirubin 10‐20 μmol/L), moderate (bilirubin 20–30 μmol/L) or severe (bilirubin > 30 μmol/L).^9^ Pulmonary involvement (leptospirosis pulmonary haemorrhage syndrome [LPHS]) was defined as disease‐causing laboured breathing, dyspnoea and haemoptysis or radiographic evidence of moderate to severe peribronchial, interstitial or alveolar infiltrates.^9^ Haemorrhagic involvement was defined as evidence of haemorrhage (other than LPHS) or the presence of disseminated intravascular coagulation (DIC) (present if all criteria are fulfilled: thrombocytopaenia, prolonged PT and aPTT).^9^, ^23^

## Results

### Patient demographics and clinical presentation

Seventeen dogs were included (Table 1). One 14‐month‐old had completed its primary vaccination course, 10 months prior to onset of clinical signs.

**Table 1:** Signalment of 17 dogs with leptospirosis

Age
Puppy <1 year; n = 2
Young adult, 1–4 years; n = 7
Middle‐aged, 5–9 years; n = 7
Geriatric, 15 years; n = 1
Median age = 4 years
Sex
Male entire, n = 6
Male neutered, n = 4
Female entire, n = 3
Female neutered, n = 4
Breeds
American Staffordshire Terrier, n = 3
Staffordshire Bullterrier, n = 2
Cavoodle, n = 2
Australian Kelpie, n = 1
Australian Shepherd, n = 1
Beagle, n = 1
Bernese Mountain Dog, n = 1
Fox Terrier, n = 1
Fox Terrier Cross, n = 1
Golden Retriever, n = 1
Greyhound, n = 1
Jack Russell Terrier, n = 1
Miniature Schnauzer, n = 1
Weight
5.7–43.1 kg (median 19.6 kg)

**Figure 1:**
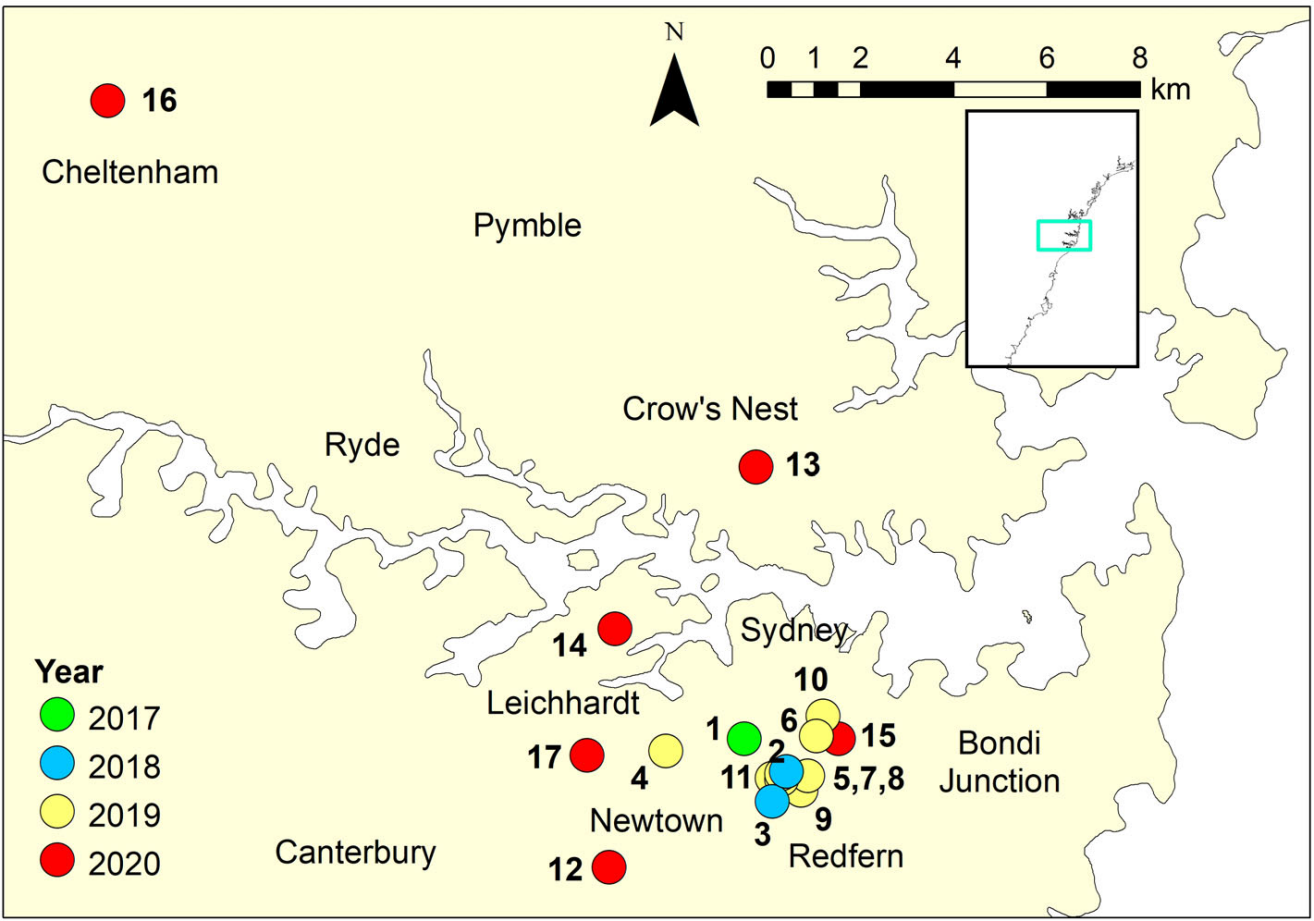
Location of 17 cases of canine leptospirosis between 2017 and 2020. 1 = Haymarket; 2, 5, 7, 8, 11 = Surry Hills; 3, 9 = Redfern; 4 = Glebe; 6, 10 = Darlinghurst; 12 = Newtown; 13 = Crows Nest; 14 = Balmain; 15 = Paddington; 16 = Cheltenham; 17 = Annandale.

Five dogs hunted rodents and one was a working sheep dog in rural New South Wales. Dogs infected between 2017 and 2019 all lived within a 3 km radius in the Inner City of Sydney. In August 2020, cases occurred in the Inner City, Inner West and Lower North Shore. In October 2020, a case was identified in the Upper North Shore. In December 2020, a case was identified in the Inner West, in a dog obtained from a farm in the Northern Tablelands (Armidale) 12 days prior (Figure 1).

Presenting complaints and physical examination findings are summarised in Table 2.

**Table 2:** Presenting complaints and physical examination findings on admission in 17 dogs with leptospirosis

Presenting complaints	Physical exam findings
Lethargy (n = 17)	Icteric mucous membranes (n = 13)
Vomiting (n = 14) Regurgitation (n = 1)	Abdominal pain (n = 12)
Inappetence (n = 14)	Dehydration (n = 11)
Diarrhoea (n = 9, haemorrhagic n = 1)	Mild lymphadenomegaly (n = 5)
Polydipsia (n = 2)	Pyrexia (T > 39°C) (n = 3)
	Hypothermia (T < 38.0°C) (n = 3)
	Increased respiratory effort (n = 2)
	Increased lung sounds (n = 2)
	Soft pulmonary crackles (n = 2)
	Peripheral oedema (n = 1) (prior to fluid therapy)

### Confirmatory tests for leptospirosis

A diagnosis of leptospirosis was confirmed by positive PCR in blood (n = 1), urine (n = 4), kidney (n = 1), blood and urine (n = 9), seroconversion (n = 2) or a positive MAT titre ≥1/800 in a nonvaccinated dog (n = 1) (Table 3).

**Table 3:** Results of PCR (n = 17, submitted 1 to 6 days after the onset of clinical signs; IDEXX or Vetnostics laboratories), Microscopic Agglutination Test (MAT; n = 12, submitted 1 to 7 days after onset of clinical signs, Queensland Health Scientific Service Cooper Plains Queensland) and histopathology (n = 8, VPDS [Veterinary Pathology Diagnostic Services – the University of Sydney] or Vetnostics laboratories) in dogs with leptospirosis

Dog number	1	2	3	4	5	6	7	8	9	10	11	12	13	14	15	16	17
PCR urine	**pos**	**pos**	**pos**	**pos**	**pos**	**pos**	**pos**	**pos**	neg	**pos**	neg	**pos**	**pos**	neg	**pos**	**pos** ^a^	neg
PCR blood	**pos**	neg	neg	neg	**pos**	**pos**	**pos**	**pos**	neg	**pos**	**pos**	neg	**pos**	neg	**pos**	**pos** ^a^	neg
PCR kidney	N/A	N/A	N/A	N/A	N/A	N/A	N/A	N/A	N/A	N/A	N/A	N/A	N/A	N/A	N/A	N/A	**pos**
Histopathology^b^	N/A	N/A	N/A	N/A	N/A	N/A	neg	**pos**	neg	**pos**	**pos**	neg	neg	N/A	N/A	N/A	neg
Serovar Arborea	N/A	N/A	N/A	<50	N/A	N/A	<50	<50	<50	<50	<50	<50	<50	<50	<50	<50	<50
Serovar Australis	N/A	N/A	N/A	**50**	N/A	N/A	**50**	<50	<50	<50	<50	<50	<50	**400**	<50	<50	<50
Serovar Bataviae	N/A	N/A	N/A	<50	N/A	N/A	<50	**200**	<50	<50	<50	<50	<50	<50	<50	<50	<50
Serovar Bulgarica	N/A	N/A	N/A	<50	N/A	N/A	<50	**50**	<50	<50	**50**	<50	<50	<50	<50	<50	<50
Serovar Canicola	N/A	N/A	N/A	<50	N/A	N/A	<50	<50	<50	<50	<50	<50	<50	<50	<50	<50	<50
Serovar Celledoni	N/A	N/A	N/A	<50	N/A	N/A	<50	<50	<50	<50	<50	<50	<50	<50	<50	<50	<50
Serovar Copenhageni	N/A	N/A	N/A	**800**	N/A	N/A	<50	**800**	**200**	**100**	**200**	**100**	<50	**1600**	<50	<50	<50
Serovar Cynopteri	N/A	N/A	N/A	<50	N/A	N/A	<50	<50	<50	<50	<50	<50	<50	<50	<50	<50	<50
Serovar Djasiman	N/A	N/A	N/A	<50	N/A	N/A	<50	<50	<50	<50	<50	<50	<50	<50	<50	<50	<50
Serovar Gryppotyphosa	N/A	N/A	N/A	<50	N/A	N/A	<50	**50**	<50	<50	<50	<50	<50	<50	<50	<50	<50
Serovar Hardjo	N/A	N/A	N/A	<50	N/A	N/A	<50	**100**	<50	<50	<50	<50	<50	<50	<50	**1600**	<50
Serovar Icterohaemorrhagiae^c^	N/A	N/A	N/A	N/A	N/A	N/A	N/A	N/A	N/A	N/A	N/A	<50	<50	**400**	<50	<50	<50
Serovar Javanica	N/A	N/A	N/A	<50	N/A	N/A	<50	<50	<50	<50	<50	<50	<50	**400**	<50	<50	<50
Serovar Kremastos	N/A	N/A	N/A	**50**	N/A	N/A	<50	**100**	<50	<50	<50	<50	<50	<50	<50	<50	<50
Serovar Medanensis	N/A	N/A	N/A	<50	N/A	N/A	<50	<50	<50	<50	<50	<50	<50	<50	<50	**100**	<50
Serovar Panama	N/A	N/A	N/A	<50	N/A	N/A	<50	<50	<50	<50	<50	<50	<50	<50	<50	<50	<50
Serovar Pomona	N/A	N/A	N/A	<50	N/A	N/A	<50	<50	<50	<50	<50	<50	<50	**100**	<50	<50	<50
Serovar Robinsoni	N/A	N/A	N/A	<50	N/A	N/A	**50**	<50	<50	<50	<50	<50	<50	<50	<50	<50	<50
Serovar Shermani	N/A	N/A	N/A	<50	N/A	N/A	<50	<50	<50	<50	<50	<50	<50	<50	<50	<50	<50
Serovar Swajizak	N/A	N/A	N/A	<50	N/A	N/A	<50	<50	<50	<50	<50	<50	<50	<50	<50	<50	<50
Serovar Tarassovi	N/A	N/A	N/A	<50	N/A	N/A	<50	<50	<50	<50	<50	<50	<50	<50	<50	<50	<50
Serovar Topaz	N/A	N/A	N/A	<50	N/A	N/A	<50	**50**	<50	<50	<50	<50	<50	<50	<50	<50	<50
Serovar Zanoni	N/A	N/A	N/A	<50	N/A	N/A	<50	<50	<50	<50	<50	<50	<50	<50	<50	<50	<50

*Note*: Boldface has been used to make positive titres more clearly visible.

^a^ Tested positive for *Leptospira* species but not *Leptospira interrogans*.

^b^ Visualisation of spiral bacteria with silver stain.

^c^ Due to changes in the reference laboratories scope of testing, samples received after July 2020 were additionally tested for serovar Icterohaemorrhagiae.

MAT results for dog 14 are 24 days after initial presentation (convalescent titre). MAT results for dog 16 are 17 days after initial MAT titre (21 days after presentation).

neg, negative; pos, positive.

Antibody titres to *Leptospira* serovars were measured by MAT in 12 dogs; seven were positive for serovar Copenhageni and one was positive for serovar Hardjo. In one dog (9) (pretreated with metronidazole), diagnosis was established by seroconversion. In one unvaccinated dog (14), diagnosis was established by a positive MAT titre of 1/800 for serovar Copenhageni, which subsequently increased to 1/1600. One (16) had positive PCR results on blood and urine to *Leptospira* species, however, this case tested negative for *Leptospira interrogans*. This dog initially had a low positive titre (1/50) to serovars Hardjo and Zanoni and subsequently seroconverted to serovar Hardjo (1/1600) 17 days later. The 14‐month‐old (15), which received the primary vaccination course had a negative MAT, likely due to insufficient time for seroconversion.

In eight cases, histopathology of the kidneys (n = 1), kidneys and liver (n = 2) or a complete necropsy (n = 5) was performed. In three, *Leptospira*‐shaped organisms were identified in the kidneys with silver stain.

### Clinicopathological findings

Haematology and serum biochemistry results were available in 15 dogs. In two retrospectively enrolled dogs, the medical record described severe azotaemia, elevated liver enzymes and icterus; however, numerical results were not recorded on the patient file. Ninety‐four percent had a mild leucocytosis with neutrophilia. A manual differential count was available in two cases, both with a mild left shift; 73% had mild to moderate thrombocytopaenia and 53% were anaemic. Anaemia was mild in most and severe in one (Table 4).

**Table 4:** Results of haematology at the time of maximal deviation from reference interval in 15 dogs with leptospirosis

	Range	Median	IQR	Reference interval
Haematocrit (%)	19–55	36	32–47.5	37–55
Thrombocytes (×10^9^/L)	31–312	90	52–218	200–600
Leukocytes (×10^9^/L)	6.7–26.3	19.3	14.7–23.0	7–12
Neutrophils (×10^9^/L)	5.6–24.2	14.8	11.9–20.0	4–9.3
Monocytes (×10^9^/L)	0.3–3.5	1.4	0.9–1.8	0.2–0.9
Lymphocytes (×10^9^/L)	0.6–2.4	1	0.8–2.05	0.9–3.6
Eosinophils (×10^9^/L)	0–0.5	0	0–0.1	0.1–1.2

IQR, interquartile range.

Biochemistry results are summarised in Table 5. All dogs developed severe azotaemia and hyperphosphataemia, 94% had increased alkaline phosphatase‐activity and hyperbilirubinaemia and 87% had increased alanine transaminase. Creatine kinase was elevated in all five cases in which it was measured. All had increased lipase and 40% an increase in amylase. Fifty‐three percent developed hyperkalaemia. Ionised calcium was measured in nine with hypocalcaemia found in five. Importantly, on initial presentation, two cases were nonazotaemic and one had a normal bilirubin.

**Table 5:** Results of serum biochemistry at the time of maximal deviation from reference interval in 15 dogs with leptospirosis

	Range	Median	IQR	Reference interval
Creatinine (μmol/L)	218–1039	621	480–799	40–120
Urea (mmol/L)	20.8–64.7	41.1	31.8–51.3	1–10
Phosphate (mmol/L)	2.4–6.0	3.4	2.9–4.7	0.8–1.6
Bilirubin (μmol/L)	8–491	245	107–446	1.2–8.1
ALT (U/L)	60–1716	196	102–527	<60
ALP (U/L)	203–3356	1215	489–1766	<110
CK (U/L)	452–4638	1521	515–3741	<200
Cholesterol (mmol/L)	2–7.2	3.4	2.6–6.0	1.4–7.5
Amylase (U/L)	662–3394	1157	671–2486	<1400
Lipase (U/L)	250–6000	1126	317–5481	<60
Protein (g/L)	36–80	58	50–62	50–70
Albumin (g/L)	20–34	24	22–29	23–43
Globulin (g/L)	16–56	32	25–43	27–44
Glucose (mmol/L)	5.2–9	6.3	5.8–6.8	3.3–6.4
Calcium total (mmol/L)	2.2–2.9	2.6	2.4–2.7	2.1–2.9
Calcium ion (mmol/L)	0.97–1.37	1.18	1.14–1.34	1.2–1.4
Sodium (mmol/L)	124–158	142	133–146	137–150
Potassium (mmol/L)	3.2–7.9	4.7	4.5–5.7	3.3–4.8
Chloride (mmol/L)	89–126	99	97–107	105–120

ALP, alkaline phosphatase; ALT, alanine transaminase; CK, creatine kinase; IQR, interquartile range.

Coagulation was measured in nine cases (Table 6). Eighty‐three percent had prolonged aPTT, and 33% had prolonged PT. Three had prolonged PT, aPTT and thrombocytopenia fulfilling criteria for DIC. One case had petechiae and thrombocytopaenia; however, PT and aPTT were not measured. Overall 24% (4/17) showed clinical or laboratory evidence of haemorrhagic involvement.

**Table 6:** Results of coagulation profile at the time of maximal deviation from reference interval in nine dogs with leptospirosis

	Range	Median	IQR	Reference interval
PT (s)	12 to >100	16	15–21	11–17
aPTT (s)	88 to >300	134	109–143	72–102

aPTT, activated partial thromboplastin time; IQR, interquartile range; PT, prothrombin time.

Urinalysis was performed in nine dogs (Table 7). Glucosuria was present in 3/9 (33%), proteinuria in 7/9 (78%), bilirubinuria in 5/9 (56%), pyuria in 4/6 (67%) and microscopic haematuria in 4/6 (67%). One had mild calcium oxalate crystalluria and one had tubular casts on sediment exam (unclassified). Urine culture was performed in six cases and was positive in one sample from the urinary catheter.

**Table 7:** Urinalysis results in nine dogs with leptospirosis

	Range	Abnormal values
USG (n = 8)	1.010–1.050	Isosthenuria (n = 3)
		Minimally concentrated^a^ (n = 4)
		Hypersthenuria (n = 1)
Glucose (n = 9)	None to 2+	Negative (n = 6)
		Trace (n = 2)
		2+ (n = 1)
Protein (n = 9)	Negative to 2+	Negative (n = 1)
		Trace (n = 3)
		1+ (n = 3)
		2+ (n = 2)
Bilirubin (n = 9)	None to large	None (n = 4)
		1+ (n = 3)
		3+ (n = 1)
		Large (n = 1)
Red cells (per HPF) (n = 6)	<5–>100	<5 (n = 2)
		20 (n = 1)
		>100 (n = 3)
Leukocytes (per HPF) (n = 6)	3–20	Neg (n = 1)
		3 (n = 1)
		<5 (n = 3)
		20 (n = 1)
Crystals (n = 6)		Ca‐oxalate (n = 1)
Casts (n = 6)		3+ (unclassified) tubular casts (n = 1)
Urine culture (n = 6)		Negative (n = 5)
		Light growth beta haemolytic *streptococcus* spp.(n = 1) – Catheter urine

^a^ Minimally concentrated: USG 1.013–1.029; hypersthenuria >1.030.

HPF, high power field; USG, urine specific gravity.

### Diagnostic imaging

Chest radiographs were taken in five dogs. In two dogs with respiratory signs, radiographic changes were consistent with LPHS (Table 8).

**Table 8:** Radiographic findings and respiratory signs in five dogs with leptospirosis (interpreted by board‐certified specialist in diagnostic imaging)

Dog number	Time radiographs taken	Respiratory signs	Imaging findings	Leptospiral haemorrhage syndrome
1	On admission	None	Marked diffuse mixed (bronchial, interstitial to alveolar) pulmonary pattern	Suspected, consistent clinical signs 2 days later and died, no necropsy
4	On admission	None	Unremarkable	Suspected, epistaxis, sublingual haematoma, no necropsy
8	On admission	Increased lung sounds and respiratory effort	Unremarkable	Suspected
9	4 days after admission	Increased respiratory effort	Diffuse mild to moderate unstructured increase in pulmonary opacity, more severe in right middle and caudal lung lobes, hazy pulmonary markings and irregular ventral margination of the lung fields, more nodular increased pulmonary opacity caudo‐dorsally	Suspected
14	On admission	None	Unremarkable	No, complete recovery

LPHS, leptospiral haemorrhage syndrome.

An abdominal ultrasound was performed in 14 dogs and findings are summarised in Table 9.

**Table 9:** Abdominal ultrasound findings in 10 dogs in which the ultrasound was performed by a board‐certified specialist in diagnostic imaging

Liver
Hepatomegaly (n = 4)
Hypoechoic parenchyma (n = 5)
Thickened gallbladder wall and common bile duct (n = 1)
Biliary sludge (n = 1)
Kidney
Pyelectasia (n = 1)
Hyperechoic renal cortex (n = 1)
Perirenal fluid (n = 6), extending into retroperitoneum (n = 3/6)
Lymphadenomegaly
Portal (n = 1)
Hepatic (n = 1)
Medial iliac (n = 3)
Mesenteric (n = 4)
Generalised (n = 1)
Other
Peritoneal effusion (n = 2)
Peritonitis (n = 3)
Mild pancreatitis (n = 1)
Mildly corrugated duodenum (n = 1)
Mild colonic wall thickening (n = 1)
Splenomegaly (n = 2)

### Organ manifestations

All dogs had renal involvement, 16 (94%) had hepatic involvement, five (29%) had pulmonary involvement and presumed LPHS and four (24%) showed bleeding tendencies (petechiae n = 1, haematemesis n = 1 and sublingual haematoma and bruising n = 1). The latter developed in a Greyhound after feeding tube placement – based on signalment; hyperfibrinolysis could not be excluded as the cause.

### Treatment regime and outcomes

Twelve dogs were treated at specialist centres and five at their general practice veterinarian.

All dogs were treated with intravenous fluid (IVF) and antibiotics. Additional supportive treatments are summarised in Table 10.

**Table 10:** Summary of drugs used for treatment in 17 dogs with leptospirosis

Treatment	Drug
Fluid therapy	IV fluids (n = 17)
Antibiotics	Ampicillin IV (n = 9)
	Amoxicillin–clavulanate IV (n = 6)
	Amoxicillin IV (n = 2)
	Cephazolin IV (n = 2)
	Doxycycline PO (n = 2)
	Enrofloxacin IV (n = 3)
	Metronidazole IV (n = 7)
Antiemetics	Maropitant IV (n = 14)
	Ondansetron IV (n = 6)
	Metoclopramide IV (n = 5, as CRI in n = 3)
Gastroprotectants	Esomeprazole IV (n = 6)
Analgesia	Buprenorphine IV (n = 9)
	Methadone IV (n = 3)
	Fentanyl IV (n = 1)
Medication to improve urine output	Frusemide bolus IV (n = 4)
	Frusemide bolus IV and CRI + mannitol bolus IV and CRI (n = 3)
	Frusemide bolus IV + mannitol bolus IV (n = 1)
	Frusemide bolus IV + mannitol CRI (n = 1)
	Dopamine CRI (n = 1)
	Noradrenaline CRI (n = 1)
Treatment for hyperkalaemia	Calcium gluconate (n = 1)
	Glucose bolus + neutral insulin CRI (n = 1)
Antihypertensive medication	Amlodipine (n = 1)
Liver protectants	S‐adenosyl‐methionine PO (n = 4)
	N‐acetylcysteine IV (n = 2)
	Ursodeoxycholic acid PO (n = 2)
Miscellaneous	Lactulose PO (n = 1)
	Vitamin K SC (n = 1)
Blood products	Fresh frozen plasma (n = 1)
	Fresh whole blood (n = 1)

CRI, continuous rate infusion; IV, intravenously; PO, per os; SC, subcutaneous.

A urinary catheter was placed in seven to measure urine output, in the remainder urine output was estimated. Five dogs were oliguric then became anuric, four were oliguric throughout, three were anuric throughout and two had normal to increased output (1.6–2.4 mL/kg/hr). One of the latter fully recovered, the other developed stage 3 chronic kidney disease (CKD). Blood pressure was measured in nine dogs. One of three hypertensive dogs (systolic blood pressure 260, 240 and 200 mmHg) was treated with amlodipine.

Seven dogs developed peripheral oedema – in the eyes (chemosis) (n = 1), periorbital (n = 1), on peripheral limbs (n = 3), face (n = 3), skin (n = 1) and neck (n = 1) or as serous nasal discharge (n = 1).

Six dogs died, nine were euthanased and two survived. Reasons for euthanasia were anuria (n = 5), respiratory distress (n = 3), seizures (n = 2), nystagmus (n = 1), refractory hyperkalaemia (n = 1) and upper airway obstruction (n = 1). Causes of death were respiratory distress, hyperkalaemia and anuria (n = 1), LPHS and seizures (n = 1), respiratory distress and LPHS (n = 1). Three died for undetermined reasons.

Of the two dogs that survived, one was hospitalised for five days and treated with IVF, antibiotics (initially amoxicillin–clavulanate 20 mg/kg intravenously three times a day, then doxycycline 5 mg/kg twice a day), antiemetics (maropitant 1 mg/kg once a day (SID)) and liver protectants (S‐adenosyl‐methionine 30 mg/kg per os SID). There was hepatorenal disease with initial IRIS AKI grade 2 (creatinine 218 μmol/L) and severe hepatic involvement (bilirubin 107 μmol/L). It was polyuric throughout hospitalisation and showed complete recovery within 1 month based on normal biochemistry results. The second dog was hospitalised for 1 week. It was treated with IVF and antibiotics (cephazolin 22 mg/kg intravenously for 1 week, doxycycline 10 mg/kg SID for 2 weeks). It had hepatorenal disease with initial IRIS AKI grade 3 (creatinine 297 μmol/L) and severe hepatic involvement (bilirubin 71 μmol/L). This dog had normal to increased urine production and developed CKD stage 3 after discharge, which is nonprogressive at the time of writing, 3 months following.

### Necropsy and histopathology findings

Necropsy was performed on five dogs and findings are summarised in Table 11; examination was limited in one given significant freeze–thaw artefact.

**Table 11:** Summary of necropsy findings in five dogs with leptospirosis

Dog number	7^a^	8	9	10	11
Jaundice	+++	+++	+++	+++	+++
Multisystemic haemorrhage (variably affecting: cutaneous, subcutaneous, lungs, kidney, gastrointestinal, heart)	−	+++	+++	++	+
Ascites	+	+++	++	++	+++
Pleural effusion	++	++	++	++	++
Pulmonary oedema	−	++	++	++	++
Hepatomegaly	−	−	+	−	+
Splenomegaly	−	−	+	−	−

^a^ Examination was limited by severe freeze–thaw artefact.

‘+, ++, +++’ mild, moderate, marked, respectively; ‘−’ not detected.

Histopathological examination of multiple organs was performed for seven, all of which included kidney (including silver staining) and liver. An additional dog only had kidney evaluated histologically. Histologic findings are summarised in Table 12.

**Table 12:** Summary of histopathological findings in eight dogs with leptospirosis

Dog number	7^a^	8	9	10	11	12	13	17
Multisystemic haemorrhage (variably affecting kidneys, lungs, gastrointestinal, heart, subcutaneous)	−	+++	+++	+++	++	++	+	++
Tubulointerstitial nephritis (lymphoplasmacytic)	++	+	+	++	+	++	++	+
Tubular degeneration +/− necrosis	−	++	+++	+++	+++	+++	++	++
Renal tubular casts (protein, cellular)	++	+++	+++	+++	++	+++	++	+
Membranous glomerulonephritis	++	+	−	−	−	−	−	−
Renal tubular and lamina spirochete organisms (silver stain, Warthin‐Starry)	NP	++	−	+	+	NP	NP	−
Hepatocellular dissociation with Kupffer cell hypertrophy	−	+++	+++	+++	+++	++	X	+++
Cholangiohepatitis (lymphoplasmacytic)	−	++	+	+	++	+	X	−
Pulmonary oedema	X	+++	+++	+++	++	X	X	+++
Pancreatitis	X	++	+	−	++	X	X	X
Lymph node follicular hyperplasia	X	++	+++	++	+	X	X	X
Cystitis	X	−	+	X	+	X	X	X
Alzheimer type II astrogliosis	X	++	−	−	++	X	X	X

^a^ Examination was limited by severe freeze–thaw artefact.

‘+, ++, +++’ mild, moderate, marked, respectively; ‘−’ not detected. ‘NP’ not performed. ‘X’ respective tissue not examined.

## Discussion

This case series describes the re‐emergence of clinical canine leptospirosis with a high case fatality rate in urban Sydney. Prior to this, canine leptospirosis had not been reported in Sydney since 1976.^21^ While disease awareness and subsequently testing increased after a leptospirosis alert was issued across Sydney, this alert was issued in July 2019 after seven cases of leptospirosis had been diagnosed by veterinarians in Sydney. Therefore, we believe that this case series represents true re‐emergence of disease and not previous under‐recognition.

Most cases of canine leptospirosis in Australia have been described in North Queensland,^4^, ^5^, ^24^ with the first report in 1940.^25^ In two case series describing 84 dogs between 1995 and 2006, antibodies to serovar Australis were most commonly identified.^4^, ^5^ Serovar Icterohaemorrhagiae was thought to be the causative serovar described in Tasmania in 1968.^26^ Reservoir hosts for serovar Australis are likely native animals, including marsupials such as bandicoots and native rats and mice, whereas the potential reservoir host for serovar Icterohaemorrhagiae is the rat.^13^, ^27^ In 1962, a seroprevalence of 7.3% was reported with serovars Icterohaemorrhagiae and Esposito being the most common.^24^ In Victoria, a seroprevalence of 10% was found in 1952 with serovar Icterohaemorrhagiae identified as the most common.^20^

The highest antibody titre determined by MAT was found for serovar Copenhageni in most cases (7/12 dogs), the potential reservoir host for serovars Copenhageni and Icterohaemorrhagiae is the rat.^13^, ^27^ MAT was performed in three out of five dogs who were known to hunt rats and serovar Copenhageni had the highest titre in all. Previously published Sydney cases provide limited information, including four Greyhounds from a kennel in 1976,^21^ five dogs with suspected leptospirosis in a boarding kennel in 1952^19^ and three dogs with suspected leptospirosis in 1952.^20^ Serovar Copenhageni was the serovar with the highest titre in most of these. Of note, cross‐reactivity between different serovars within the same serogroup (e.g. Icterohaemorrhagiae and Copenhageni) can occur and not all serovars were tested in all studies. In serosurveys of dogs in 2004,^16^ 1990^17^ and 1972,^18^ serovar Copenhageni was the most prevalent. Therefore, prior to the current cluster of cases, the monovalent vaccine containing bacterins of *L. interrogans* serovar Copenhageni (Protech C2i) has been used in dogs in New South Wales if deemed necessary, based on the knowledge of these prior cases. Based on these studies, the Sydney dog population might have been free from disease during the past several decades; however, exposure and subclinical infection are evident.

One dog showed seroconversion for serovar Hardjo, the reservoir hosts of which are sheep and cattle and this dog was used for herding in regional New South Wales (Young, Richmond, Hawkesbury). Clinical cases of leptospirosis showing an increase in antibody titre to serovar Hardjo are rarely reported with only two cases described in Queensland^4^, ^5^ and five in the USA^11^, ^12^, ^28^, ^29^ and none detail information about contact with reservoir hosts. The seroprevalence of Hardjo in dogs has previously been estimated to be low in New South Wales with 0.5% in 1972,^18^ 0.4% in 1990^17^ and 0% in 2004,^16^ however, extensive recent seroprevalence studies have not been published.

Predicting the infecting serovar based on a single MAT result is problematic due to serologic cross‐reactions, especially in acute stages^30^ and ideally, a MAT titre should be repeated in 7–14 days. This was not always performed due to early fatalities or financial constraints. In most previous studies, only 6–8 serovars were included in the MAT panel. In our study, the panel contained 23 serovars, increasing sensitivity in detecting infection and standardly applied to all cases of suspect leptospirosis in humans and animals in Australia, via the WHO reference laboratory. Definitive identification of the causative serovar requires culture, which is technically difficult and takes several months, which is impractical in the clinical setting.^1^

Diagnosis was confirmed with PCR testing of blood or urine in most dogs and PCR testing of kidney tissue in one, whereas diagnosis in previous studies was based on clinical presentation and antibody testing.^4^, ^5^, ^19^, ^20^, ^21^ Samples for PCR should be collected prior to antibiotic administration.^2^, ^3^ Although several studies have shown positive urine PCR results in healthy dogs (shedders),^31^, ^32^, ^33^, ^34^, ^35^, ^36^ a positive result in a dog with consistent clinical signs and clinicopathologic changes suggest leptospirosis.^1^ False‐negative PCR can be encountered due to low bacterial loads or after administration of antimicrobials.^1^ False positive PCR results could occur due to contamination of the sample, which was considered unlikely in our cases due to the preventative measures in the reference laboratory setting. The positive PCR for *Leptospira* species but inability to detect *L. interrogans* in one case could be explained by very low levels of DNA. Infection with serovar Hardjo was later suspected following seroconversion. In another dog, PCR was negative in blood and urine, however, positive on kidney tissue. This could also be explained by low levels of shedding. In another two dogs, PCR results in blood and urine were also negative. One had been treated with metronidazole; the other did not receive antibiotics prior to presentation. The untreated dog with negative PCR had a high MAT titre to serovar Copenhageni of 1/800 initially with subsequent increase to 1/1600. This dog was seen carrying a rat 10 days prior to presentation. The negative PCR and full recovery of this dog may be due to exposure to a lower number of organisms. Metronidazole is not described for treatment of dogs with leptospirosis, but it cannot be excluded that administration resulted in a false‐negative PCR. This dog initially had negative MAT titres and developed a positive titre of 1/200 against serovar Copenhageni 4 days later. Subsequent MAT testing could not be performed.

Possible explanations for development of leptospirosis despite vaccination, which occurred in one dog, includes host factors leading to an inadequate immune response, vaccination failure or infection with a serovar other than serovar Copenhageni. It appears currently available vaccines elicit serogroup‐specific immunity and partial immunity to heterologous serogroups.^2^

Clinical signs and physical exam findings were similar to those previously described.^5^, ^6^, ^7^, ^8^, ^10^, ^11^, ^37^ Icteric mucus membranes were detected in a higher proportion of dogs in our study (76%) compared to what has been previously described (10%–45%).^6^, ^7^ Hyperbilirubinaemia (94%) was more common in our patients compared to what has been described in dogs overseas (17%–81%);^7^, ^38^ however, similar to dogs in Queensland.^5^ Based on severity of hyperbilirubinaemia, hepatic involvement was classified as severe in all affected dogs. Liver involvement has been strongly associated with a negative outcome in a previous study.^9^ While pancreatitis is a known complication in canine leptospirosis and could cause cholestasis,^3^, ^37^ mild pancreatitis (without any evidence of bile duct obstruction) was only found in 1/10 patients where abdominal ultrasound was performed, hence contribution of bile duct obstruction due to severe pancreatitis is unlikely.

All dogs had renal involvement, but two were nonazotaemic at initial presentation. Therefore, renal parameters should be rechecked within 24 h following initial testing, in nonazotaemic dogs with consistent clinical signs and known risk factors. Rechecking renal parameters every 48 h ongoing is recommended while hospitalised. Reports of leptospirosis without renal involvement are extremely rare in the literature.^9^, ^12^, ^37^ Hyperkalaemia is a common complication of anuric/oliguric kidney failure and can cause severe bradyarrhythmias and cardiac arrest. Electrolytes should be checked at least twice daily to adjust fluid therapy. Treatment of severe hyperkalaemia was indicated in two dogs. Haemodialysis would have been helpful for these patients however was not available in New South Wales until 2021.^11^

Thrombocytopaenia was present in 73% and is commonly found in dogs with leptospirosis. Proposed mechanisms include vasculitis due to circulating leptospires causing endothelial injury with subsequent platelet adhesion and activation of the coagulation cascade,^11^ DIC,^6^, ^7^ immune‐mediated destruction^39^ or splenic sequestration.^6^ Results were consistent with DIC in three – all showed bleeding tendencies. Early aggressive treatment and supportive care are important to counteract development of DIC. Transfusion with fresh frozen plasma is recommended in dogs with DIC and signs of bleeding.^2^

In dogs with LPHS, typical findings on thoracic radiographs include an interstitial (mild), reticulonodular (moderate) and alveolar (severe) lung pattern.^6^ Radiographs are recommended even in the absence of respiratory signs to detect early lesions. Preventative measures include avoidance of stress, overhydration/hypervolaemia and control of systemic hypertension.^2^ Radiographic changes consistent with LPHS were found in two dogs in our study. Overall, 29% had pulmonary involvement; however, radiographs were not taken in all patients at the time of respiratory distress. In other studies, pulmonary involvement has been described in up to 70%.^6^, ^9^ Severe lung involvement is associated with high mortality.^8^ The pathogenesis of LPHS is unknown, however, systemic inflammatory, immune‐mediated and direct leptospiral effects have been proposed.^40^ Treatment includes supportive care, oxygen therapy and in severe cases mechanical ventilation. Treatment with glucocorticoids, bronchodilators (theophylline) and frusemide has been attempted in earlier studies, but improved outcome was not demonstrated,^6^ and further studies are needed before recommending these treatments.^2^

The high fatality rate compared to published cases^6^, ^8^, ^11^ could be explained by multiple factors. First, the urban Sydney dog population is considered immunonaïve with low reported levels of exposure,^16^ therefore, more susceptible to infection. Second, it is possible that a more virulent strain of serovar Copenhageni is involved. Third, infection rates have risen during an episode of drought. This implies direct contact with reservoir hosts and inoculation with high numbers of organisms might be a more likely source than contact with contaminated soil or water. Finally, oliguric and anuric kidney failure was present in 13 dogs. Haemodialysis improves outcomes in dogs with leptospirosis^11^, ^28^ but unfortunately was not available in New South Wales.

In all but one dog with positive MAT titres, the highest was detected for serovar Copenhageni, however, in many dogs antibody titres were not measured or were negative likely due to insufficient time for seroconversion. This, and fatal leptospirosis in a vaccinated dog raises concern whether the currently available vaccine containing bacterins of *L. interrogans* serovar Copenhageni (Protech C2i) is protective in the current outbreak. Future studies are needed, including ongoing investigation of new leptospirosis cases, seroprevalence in the Sydney dog population prior to the current outbreak and characterisation of the immune response after vaccination to determine duration of immunity and presence of cross‐protection against other serovars.

## Conflicts of interest

The authors declare no conflicts of interest for the work presented here.
